# Effect of Platelet-Rich Fibrin Application on Non-Infectious Complications after Surgical Extraction of Impacted Mandibular Third Molars

**DOI:** 10.3390/ijerph18168249

**Published:** 2021-08-04

**Authors:** Grzegorz Trybek, Justyna Rydlińska, Magda Aniko-Włodarczyk, Aleksandra Jaroń

**Affiliations:** Department of Oral Surgery, Pomeranian Medical University in Szczecin, 72 Powstańców Wlkp. St., 70-111 Szczecin, Poland; justyna.rydlinska@pum.edu.pl (J.R.); dominika.wlodarczyk@pum.edu.pl (M.A.-W.); jaronola@gmail.com (A.J.)

**Keywords:** third molar, impacted third molar, mandibular third molar, impaction, PRF, platelet-rich fibrin, complications

## Abstract

Due to the frequent development of non-infectious complications after surgical removal of the third lower impacted tooth, many techniques are used to reduce their severity. Among them is the technique of applying platelet-rich fibrin to the post-extraction alveolus. The study included 90 consecutively enrolled patients. Eligible patients were randomly assigned to two groups: patients with and without platelet-rich fibrin introduced into the postoperative alveolus. Pain, swelling, trismus, and temperature were evaluated after the procedure. Pain intensity was significantly higher in the control group than in the study group at 6 h, 1, and 3 days after surgery. PRF application did not significantly affect the intensity of swelling. Body temperature was significantly higher in the control group than the study group on day two after surgery. The trismus was significantly higher in the control group than in the study group at one, two, and seven days after surgery. Application of the PRF allows for a faster and less traumatic treatment process. It will enable for speedier recovery and return to active life and professional duties.

## 1. Introduction

Statistically, wisdom teeth are most often impacted, which is considered a pathological condition. The late formation of third molars and the evolution of the size of the mandible resulted in insufficient space for proper eruption. Differences in the type of food consumed and physical activity result in a reduction in jaw size [1,2]. Genetic factors are also taken into account [3,4]. Lack of sufficient space for the eruption of the third molar is not infrequently manifested by pain, periradicular inflammation, root resorption of adjacent teeth, and cyst formation [2,5]. Surgical removal of third lower molars, like any surgical procedure, could be associated with postoperative complications [6]. According to the study of Barbosa-Rebellato et al., postoperative complications are more frequent after the removal of third molars located in the mandible (*p* = 0.006) than in the maxilla [6]. This is related to the higher density of the cortical bone plate in this region. The age between twenty-four and thirty years is considered the best age for surgical removal of wisdom teeth due to optimal bone elasticity, regenerative potential, and blood supply [7,8]. The relationship between patient gender, operator experience, and incidence of complications is a debatable issue in the literature [9]. Blondeau et al. observed more frequent healing problems after surgery among female patients, although the differences were not statistically significant [7]. After the lower wisdom teeth removal, complications are qualified in the literature as-immediate postoperative tissue reactions and postoperative complications. Some authors refer to the physiological consequences of surgical tooth extraction as non-infectious complications, including postoperative pain, swelling, trismus, lymphadenopathy, and elevated body temperature [10,11]. These are the result of postoperative tissue inflammatory response. To improve the recovery period after surgical removal of lower wisdom teeth, a variety of pharmacological and non-pharmacological methods are used [12]. Complications that require immediate implementation of treatment are referred to as primary complications. These include abscess, mandibular fracture, cases of uncontrolled bleeding, neurological damage. The need for intervention when these occur is often associated with irreversible changes. Secondary complications are defined as those that resolve spontaneously: pain, swelling, and trismus [13].

Due to the frequent development of non-infectious complications after surgical removal of the third lower impacted tooth, many techniques are used to reduce their severity. Among them is the technique of applying platelet-rich fibrin to the post-extraction alveolus. The effect of this method on the non-infectious complications after surgical removal of the lower wisdom tooth is currently debated in the literature. Platelet-rich fibrin-PRF-belongs to a new generation of platelet concentrates. As an autologous material, it is widely used in dentistry to promote healing and tissue regeneration. Due to its repair potential, PRF is used in [14,15,16,17]:

- Dental surgery-as a restoration of bone defects, filling of post-extraction alveoli, guided bone regeneration procedures, closure of oro-auricular junctions, procedures to raise the floor of the maxillary sinus;
- Periodontology-treatment of recessions, treatment of bone defects, treatment of periapical lesions;
- Developmental dentistry-pulpotomy procedures, revascularization, apices.

The surgical removal of lower wisdom teeth is a commonly performed surgical procedure. Extraction of these teeth is often accompanied by non-infectious complications [18]. The disparity of data in the literature on the effect of platelet-rich fibrin application on the severity of complications after surgical removal of lower impacted wisdom teeth inspired us to conduct our study. The study aimed to evaluate the effect of platelet-rich fibrin applied to the alveolus after surgical removal of the impacted lower wisdom tooth on the intensity of pain, the amount of swelling, the level of trismus, the body temperature, and the area of surgery.

## 2. Materials and Methods

The study was conducted in the Department of Oral Surgery of Medical University after obtaining the consent of the Bioethics Committee with the number KB -0012/37/18.

### 2.1. Baseline Characteristics

The study included 90 consecutively enrolled patients: 62 females and 28 males aged 18–37 years with indications for surgical removal of wisdom teeth in the mandible. Patients who were eligible for the study were generally healthy, non-smokers, with no local or general inflammation symptoms. The following patients were excluded: patients under eighteen years of age, who did not have to undergo bone reduction during surgery, women in the periovulatory period, during menstruation, pregnancy, and lactation, with an allergy to clindamycin and with WBC, ESR, CRP values indicating ongoing inflammation. Eligible patients were assigned to groups by convenience sampling:

Study group (*n* = 45)—patients with platelet-rich fibrin introduced into the postoperative alveolus.

Control group (*n* = 45)—patients who did not receive postoperative platelet-rich fibrin injection.

Before surgery, patients were informed about the study and asked to fill out a written consent for the procedure and participation in the study.

### 2.2. Preparation of Platelet-Rich Fibrin

In patients enrolled in the study group, venous blood was collected into two sterile 10-mL vacuum tubes before the procedure. After collection, the material was immediately subjected to centrifugation in an EBA-200 (Hettich, Germany) for 12 min at 2700 rpm. As a result, a centrifuged three-layer structure was formed in the tubes, of which the middle layer was platelet-rich fibrin. The PRF was separated from the other layers 2 mm below the point of contact with the lower red blood cell layer to preserve the remaining platelets, usually located below the interface between the two layers. The PRF formed in the two tubes was arranged in the post-extraction cavity in two layers. The inner layer was formed by plasticized PRF from the first tube, inserted directly into the site of the post-extraction cavity. The second outer layer of PRF, derived from the second tube, was molded into a PRF-box by gentle compression with a loading plate. A fibrin membrane of uniform thickness was thus obtained, covered with the first layer of PRF previously introduced into the alveolus.

### 2.3. Surgical Technique for Removal of Impacted Lower Third Molars

Each patient 60 min before surgery received a single oral dose of 600 mg of clindamycin. All surgical removal of the lower wisdom tooth was performed according to standard procedure, by one doctor, an oral surgery specialist with extensive clinical experience. The procedure was performed under local anesthesia-regional and intrathecal. Lidocaine 2% with noradrenaline 0.00125% (WZF Polfa S.A., Warsaw, Poland), in the amount of three 2 mL ampoules. Exposure of the area of the impacted lower molar was then performed, forming a full-thickness triangular flap. Removal of the alveolar bone cover of the mandibular alveolar process was performed with a drill-the crown-root separation was performed if necessary. Using Bein’s straight lever and/or Meissner’s forceps, the impacted third molar was removed. In patients in the study group, PRF was inserted bilaterally into the cleaned post-extraction defect. The flap was then repositioned, and the resulting wound was closed with knotted sutures for seven days. After surgery, the patient maintained a sterile gauze tampon in the operated area for twenty minutes. Each participant was advised to use a rinse with a 0.1% chlorhexidine solution three times a day and take oral ketoprofen 100 mg-one tablet twice a day for three days. In addition, patients were required to follow a semi-liquid diet and exercise restriction seven days after surgery.

### 2.4. Methodology of Evaluation of Complications after the Removal of Impacted Lower Third Molar

Swelling, trismus, and temperature were measured for each patient. All parameters were assessed before surgery and at follow-up visits on postoperative Days 1, 2, and 7.

#### 2.4.1. Measurement of Swelling

**Figure 1:**
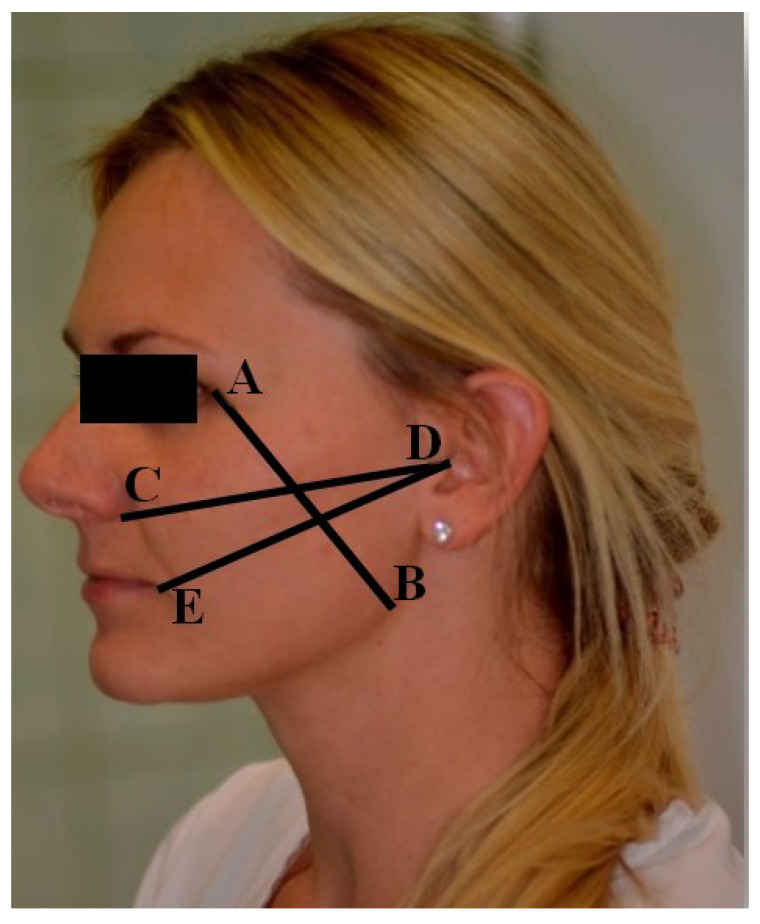
Measurement lines and points on the face.

Soft tissue edema was examined by measuring three lines delineated on the skin of the operated side by six skin landmarks (Figure 1):

- Vertical line AB determined by Point A—the lateral angle of the eye exocanthion (Ex) and Point B—the point of the angle of the mandible gonion (Go);
- Horizontal line CD delimited by Point C—the most lateral on the wing of the nose point—alare (Al), and the most distal point of the ear scrape, skin Point D—tragus (T);
- Horizontal line DE defined by Point D, the most proximal point of the earlobe tragus (T), the skin point, and the corner of the mouth, Point E—cheilon (Ch).

#### 2.4.2. Trismus Intensity Measurement

The magnitude of potentially existing trismus was assessed using a certified digital caliper (OTMT, New York, NY, USA) with a scale accurate to 0.01 mm. In each patient, the intermaxillary distance between the incisal edges of the upper and lower right central incisors was measured at the possible maximum jaw opening, according to the scenario of Ustün et al. [19].

#### 2.4.3. Measurement of Pain Intensity

Pain assessment was measured using the Numerical Rating Scale (NRS), developed by Downie et al. [20], which contains 11 pain intensity grades from 0 to 10, where 0 means no pain at all and ten means the worst imaginable pain. Patients were asked to rate their intensity at six and twelve hours after surgery and postoperative Days 1, 2, 3, 4, 5, 6, and 7.

#### 2.4.4. Body and Perioperative Site Temperature Measurement

Patients were evaluated for the operated area, non-operated area, and body temperature. Perioperative, non-operative, and body temperatures were measured before tooth extraction and postoperative Days 1, 2, and 7 at regular times using a WelchAllyn SureTemp Plus 690 (USA) thermometer. Temperature measurements of the operated and non-operated region were performed with the patient’s mouth closed, and the probe placed intraoral. The probe with a disposable sheath was placed in the retrodental spatium. Body temperature was measured in the patient’s axillary fossa, where the probe was placed at the highest possible point. Surrounding tissues surrounded the probe’s tip; the patient’s arm was pressed against the body and kept in this position until the end of the measurement.

### 2.5. Methodology of Statistical Analysis

Statistical analysis was performed in R software, version 3.5.1 (R Foundation for Statistical Computing, Vienna, Austria, 2017). Standard measures of location and measures of variability were used to describe quantitative variables. Calculations of the arithmetic mean, standard deviation, median, and quartiles were performed. A *p*-value of 0.05 was taken as the level of significance. All *p*-values that were below 0.05 were interpreted as indicating significant relationships. The normality of the distribution of the variables was tested using the Shapiro–Wilk test.

To characterize the study sample, the values of qualitative variables in the groups were compared using the Chi-square test (with Yates correction for 2 × 2 tables).

Comparison of the values of quantitative variables in two groups was performed using the Student’s *t*-test (when the variable had a normal distribution in these groups) or the Mann–Whitney test (otherwise).

## 3. Results

### 3.1. Baseline Characteristics

The study included 90 subjects: the study group (*n* = 45) had 29 women, and 16 men, the control group (*n* = 45) included 33 women and 12 men. The mean age in the study group was 26.16 ± 5.85, and in the control group, 26.09 ± 7.04. The data are presented in detail in Table 1, Table 2 and Table 3.

### 3.2. Comparative Analysis of Pain Intensity after Surgical Removal of Impacted Lower Wisdom Tooth at Six and Twelve Hours after Surgery and Postoperative Days 1, 2, 3, 4, 5, 6, and 7 between the Study and Control Groups

Patients in both groups presenting for the procedure reported no pain on the tooth scheduled for extraction. Six hours later and on postoperative Days 1 and 3, the pain level differed significantly (*p* < 0.05) between the patients in the study and control groups. A significant difference in pain sensation occurred on a postoperative Day 1. Participants in the control group felt pain with greater intensity. A significant difference in pain level was observed on postoperative Day 3. The values of the pain level of the study and control groups, as measured by the NRS scale, at each time point are shown in Table 4.

### 3.3. Comparative Analysis of Jaw Opening Size before Surgery and 1st, 2nd, and 7th Postoperative Days between the Study and Control Groups

The analysis of trismus size was performed by measuring the intermaxillary distance on postoperative Days 1, 2, and 7. The study and control groups differed significantly (*p* < 0.05) in the size of maxillary dilation after Days 1, 2, and 7. In the study group compared to the control group, significantly less jawbone was recorded on postoperative Days 1, 2, and 7 (Table 5).

### 3.4. Comparative Analysis of Soft Tissue Edema Measured by Lines: AB, CD, DE before Surgery and on Postoperative Days 1, 2, and 7 between the Study and Control Groups

The length of the AB line, measured between the exocanthion (Ex)–gonion (Go) points before surgery, and at the follow-up visits on Days 1, 2, and 7, was not significantly different between the groups (*p* > 0.05). The length of the CD line measured between the alare (Al) and tragus (T) points before surgery and at the follow-up visits on Days 1, 2, and 7 were not significantly different between the study groups (*p* > 0.05). The length of the DE line measured between the tragus (T) and cheilon (Ch) points before surgery and on Days 1, 2, and 7 were not significantly different between the groups (*p* > 0.05). Detailed data are shown in Table 6.

### 3.5. Comparative Analysis of Body and the Operated Site Temperature in the Study and Control Groups Measured before Surgery and on Postoperative Days 1, 2 and 7

The results of body temperature measurements among the patients of the study and control groups before surgery showed no significant differences (*p* = 0.196). On postoperative Day 2, significant differences (*p* = 0.022) in body temperature values were observed. The comparative analysis of the mean values of the temperature of the operated side among the patients of the study group and the control group both before surgery and on the 1st, 2nd, and 7th postoperative days showed no significant differences (*p* > 0.05). Detailed data are shown in Table 7.

## 4. Discussion

The surgical procedure for removing mandibular third molars is associated with decreased quality of life during the recovery period [21,22,23]. One method to reduce the severity of postoperative discomfort is the use of platelet-rich fibrin. Platelet concentrates, as autologous, biologically active preparations, are currently used in many areas of medicine to improve tissue healing through the release of growth factors [24]. Platelets contain many factors that play an essential role in stimulating cell proliferation and neovascularization. Platelet-derived growth factor, vascular endothelial growth factor, and transforming growth factor β1 play key roles [17]. Many papers in the literature describe different generations and types of platelet concentrate to promote wound healing [25,26,27]. The disadvantages of these biomaterials are the risk of causing allergy and the relatively short period of release of growth factors—about three days [25,28]. There is a lot of divergent information in the literature regarding the therapeutic effect of PRF infused into the post-extraction alveolus [2,29,30,31]. The topic of platelet-rich fibrin application on the temperature of the operated area and body after a lower wisdom tooth extraction is completely ignored. The blood clot, which forms spontaneously within hours after tooth extraction, platelet-rich fibrin is more stable and easily formed. This results in adequate coverage of exposed nerve endings in the alveolus and reduced pain. Platelet-derived growth factor PDGF aids the restoration of vascular integrity and wound coverage, transforming growth factor-beta TGF-β1, epidermal growth factor PD-EGF, insulin-like growth factor IGF-1 released from the fibrin matrix [32]. There are many factors during the surgical procedure that influence the intensity of the trismus after removal of a third lower molar. These factors include individual differences in the anatomy of the temporalis muscle as well as iatrogenic factors. Sometimes, effective removal of the third molar is hindered by the lowest part of the temporalis muscle tendon location. In this case, it is necessary to cut the tendon to have full access to the distal and buccal parts of the bone covering the impacted tooth. In addition, the method of flap retraction affects the intensity of postoperative com-plications. Maintaining excessive tension on the flap with a retractor causes periosteal damage. Placing the retractor too deep on the buccal side, beyond the area of the external oblique crease, results in increased swelling, pain, and reduced range of motion. Injury to the muscle stimulates a reflex contraction caused by pain, which in the literature is referred to as “muscle guarding.” This is known as muscle guarding and is manifested by trismus. This is considered to be an innate analgesic mechanism [13,33]. The local tissue response to injury is acute inflammation. The main clinical manifestations are reflected in vascular flow, increased permeability, and migration of leukocytes to the site of injury. In addition to the local tissue response, systemic and metabolic changes are also observed [34,35]. Temperature, therefore, plays an essential role in the proper functioning of every system within the body. Despite environmental temperature changes, humans maintain a relatively constant body temperature of 37 ± 0.5 °C [36]. Internal body temperature is regulated by a center located in the hypo-thalamus and spinal cord [37]. Body temperature is one of the parameters of ongoing inflammation. There is no information in the literature regarding the effect of PRF application on the temperature value of the wound area. The observed changes between groups may be due to the release of endogenous pyrogens from platelet-rich fibrin. Endogenous pyrogens, i.e., substances causing fever, include IL-1, IL-6, TNF-α [38,39,40]. Besides, PRF is characterized by properties that support angiogenesis and bone regeneration. The increased content of cytokines that help the healing process and being temperature-inducing factors enhance local blood circulation. Increased temperature in acute inflammation by improving regional blood flow accelerates chemotaxis and phagocytosis and increases tissue oxygenation. This creates a suitable environment for bone and soft tissue formation processes [41,42].

Monitoring the local temperature in the operated area after applying PRF to the post-extraction alveolus is a novel method that allows a more precise assessment of the standard healing period. In our study, we clinically observed an increase in the temperature of the operated area after the application of platelet-rich fibrin, which was accompanied by a decrease in the intensity of postoperative complications.

In the literature, conflicting positions can be observed on the effect of platelet-rich fibrin, applied to the alveolus, on pain intensity after surgical removal of third lower molars [2,30,31,43,44,45,46,47]. In all the discussed articles, the Visual Analogue Scale (VAS) was used to assess pain. According to the studies of Thong et al. [48], Hjermstad et al. [49], Fereira-Valente et al. [50], VAS and NRS scales can be used interchangeably due to the high correlation of the scores. In the present study, patients who reported no pain on the third lower molar (NRS = 0) were eligible to participate. Patients who had platelet-rich fibrin injected into the alveolus after third lower molar extraction reported significantly less pain after six hours and on days 1 and 3, compared to participants in the control group. Considering that before surgery, the NRS value in all patients was 0, it can be concluded that the rate of increase in pain was faster in the control group participants. Different results from our work in the same time interval, i.e., after six and twelve hours, were obtained by Asutay et al. [45] and Gülşen and Şentürk [46]. In Asutay’s study, no differences in patients’ pain perception were observed after both six and twelve hours [45]. In Gülşen and Şentürk’s [46] study, similar pain intensity results were obtained among the control and study groups as in Asutay’s work [45]. The authors’ study, which did not obtain significant differences in pain perception in patients who had PRF inserted into the post-extraction alveolus, followed a different pattern from our work [30,44,45,46]. Asutay et al. studied thirty patients who had 60 lower wisdom teeth removed bilaterally in mesial-angular alignment [45]. Surgical procedures were performed at 4-week intervals. Gülşen and Şentürk also enrolled thirty patients with the need for bilateral extractions to participate in the study [46]. The extraction procedure was performed on third lower molars in the vertical position, class IB according to Pell and Gregory. Our study showed a more significant variation in the spatial position of teeth scheduled for extraction than in the above investigators. In addition, root anatomy, which is sometimes independent of the spatial position of the tooth, is an essential factor determining the difficulty of the procedure [51]. It should be noted that variations in the root structure of wisdom teeth often affect the anatomic scope of the surgical extraction procedure. Bone removal and the need to dissect the tooth significantly affect complications [6]. The angle of the second molar, the development of the dental follicle, the shape of the root, and the height of the mandibular body in the area of the wisdom tooth to be removed are factors that determine the difficulty of the procedure [52]. Therefore, it is not possible to verify whether all procedures performed by Asutay et al. [45] and Gülşen and Şentürk [46] were of equal difficulty. According to WHARFE, which were used in our work, the criteria allow a more precise preoperative assessment of the difficulty of third lower molar removal. Besides, the studies of Asutay et al. [39] and Gülşen and Şentürk [46] were conducted on a relatively small group with a total of 60 extractions. Özgül et al. performed bilateral surgical extraction of third molars in the mandible among fifty-six patients [30]. Pain intensity, tested separately for alveoli with and without PRF inserted, on day 1, day 3, and day 7, was not statistically different between the two sides. The reliability of the recorded pain sensations is questionable due to the scenario of the procedures. The study authors performed surgical procedures on the control and study sides during one visit. This may have created difficulty for pa-tients in accurately distinguishing postoperative pain intensity for each side. The positive effect of PRF in reducing pain intensity after surgical removal of third impacted tooth was observed by Bilginaylar K., Kumar et al., Daugela et al. [2,31,53]. Kumar et al. conducted a study on thirty-one patients [2]. The pain was assessed using the VAS scale according to Pasqualini [54], using only 4, following terms: none, mild, light, severe. Authors Kumar et al. conducted a short observation of the intensity of post-extraction pain—one day [2]. In this case, it is not possible to verify the rate of increase and/or decrease of pain levels in all patients. Besides, the scale used contains a narrow spectrum of descriptions depicting pain, making it less accurate than the numerical NRS scale in our study. An interesting study on the control of non-infectious complications was conducted by Bilginaylar K. et al. [31]. The authors compared the effects of piezosurgery along with PRF application, PRF application, and conventional rotary devices on the level of postoperative pain, trismus, and swelling. The use of PRF reduced postoperative pain sensations in patients, and in addition, the use of the piezosurgery technique resulted in a reduction in the intake of analgesics.

The development of edema occurs within 48 to 72 h after surgical removal of the lower wisdom tooth [26,55,56]. It is essential to have an adequate observation period to assess its accrual on subsequent postoperative days reliably. The results of Özgül et al. [30] and Kumar et al. [2] showed a significant effect of platelet-rich fibrin on reducing swelling after surgical removal of the impacted lower third molar. Özgül et al. measured after the 1st, 3rd, and 7th postoperative days using a flexible ruler [30]. They observed significantly less swelling in the horizontal dimension on postoperative days 1 and 3 on the side with fibrin inserted (*p* < 0.05) than the control side, which was a conventionally sutured alveolus. The study’s methodology may be questionable due to the comparison of the amount of swelling between simultaneously operated sides. Due to physiological facial asymmetry in the length of vertical and horizontal lines before surgery may have shown differences, which was not considered in the study. As well as the postoperative period and the associated food intake, the predominance of chewing one side or the position of the head during sleep. The order of the mandibular impacted tooth removal procedure, which was not described, may also have influenced the result obtained by the authors. In this case, a more significant increase in swelling would have occurred on the side operated on first. Kumar et al. evaluated swelling on an only 4-point scale, according to which no swelling or mild, mild, severe tissue swelling after lower third molar removal procedure was noted. In this study, mild tissue edema was more frequently observed on a postoperative day among patients in the study group than patients in the control group [2]. The method used to verify the amount of swelling less accurately reflects the swelling of the tissues after surgery than measuring the length using the tape method, according to Gabek and Matsumara or 3D digital facial imaging and comparison of three-dimensional meshes by overlaying and comparing them using colour maps [57]. The short follow-up period of swelling in patients undergoing surgical removal of a lower impacted wisdom tooth is questionable. In the study of Kumar et al., it is only the 1st day [2]. 

In the present study, the value of the measured intermaxillary distance was significantly different on day 1, day 2, and day seven between the groups. A similar effect of platelet-rich fibrin after insertion into the post-extraction alveolus on day one was observed by Kumar et al. [2]. The authors additionally recommended jaw physiotherapy to the patients. Additional muscle exercises performed during the recovery period may significantly affect muscle relaxation, resulting in decreased trismus. In addition, the authors did not observe the jaw compression on postoperative days, so it is not possible to compare the effect of platelet-rich fibrin on the rate of jaw opening recovery with the preoperative state. The results obtained in the study of Asutay et al. [45], Gürler et al. [44] differ from our results and those of the authors of the mentioned publications. Based on statistical analysis, the researchers showed that the application of PRF did not significantly reduce the intensity of trismus.

There are a negligible number of articles in the literature on the observation of body temperature during the first days after surgical removal of lower third molars and other surgical procedures. This topic was addressed in the context of the appropriateness of antibiotic therapy before as well as after wisdom tooth extraction by Kaczmarzyk et al. [58], Calvo et al. [35], Milani et al. [59].

Milani et al. conducted 80 surgical wisdom tooth extractions in healthy patients using a randomized three double-blind trial in which patients were given a placebo and an antibiotic before and after the procedure [59]. Body temperature was observed before surgical intervention and on days 4 and 7 after surgery. An earlier study by Kaczmarzyk et al. was also based on a double-blind design [58]. However, body temperature measurements were taken as early as the day after surgery and on days 2 and 7. Calvo et al. conducted a study on 110 patients who did not receive an antibiotic [35]. The change in body temperature was observed on the 2nd and 7th days after surgery. Similar to our study, all authors reported stable body temperature distribution during the postoperative observation period, and slight differences between the groups were not clinically significant. The temperature of the operated area can be assessed by determining its absolute value—thanks to the thermodynamic observation of the wound temperature or body temperature—and relative to the surrounding tissues [60,61,62,63]. In our study, the temperature of the operated area was measured using a digital thermometer, the probe of which was placed in the interdental space, which is the area of the surgical procedure performed. The increase in temperature, compared to preoperative values, on each day was higher in patients who received platelet-rich fibrin after removal of the lower third molar. 

The results of this study are clinically relevant. The development of a method that can prevent pain, swelling, or trismus allows for a faster and less traumatic treatment process. The occurrence of non-infectious postoperative complications affects the patient’s quality of life. It will enable for speedier recovery and return to active life and professional duties. The material—PRF is autogenous, easy to obtain, cheap, does not cause allergic reactions or rejections, making it well accepted by patients. The study has some limitations. The study group consisted of only 90 patients. In addition, both temperature, pain, swelling, and trismus were not measured continuously but once a day. In our study, after the application of platelet-rich fibrin, no reduction in swelling was observed on days 1, 2, and 7 after the lower third molar extraction. However, further studies under isolated conditions allowing the elimination of variable physical activity of patients after lower wisdom tooth extraction should be considered. This study shows that various methods of edema measurement can potentially affect the results obtained. The type of flap used and the patient’s physical activity during this period may also influence the recovery period. In our study and the authors mentioned above, the patient’s behavior after removing the lower third molar was not monitored. Furthermore, the method of examining trismus and swelling could be more precise, e.g., by using 3D digital facial imaging and comparison of three-dimensional meshes by overlaying and comparing them using color maps. We want to conduct another study on a more extensive study group using the 3D scanning method to evaluate edema.

## 5. Conclusions

Pain intensity was significantly higher in the control group than in the study group at 6 h, 1, and 3 days after surgery. PRF application did not significantly affect the intensity of swelling. Body temperature was significantly higher in the control group than the study group on day two after surgery. The trismus was significantly higher in the control group than in the study group at one, two, and seven days after surgery. Application of the PRF allows for a faster and less traumatic treatment process. It will enable for speedier recovery and return to active life and professional duties.

## Figures and Tables

**Table 1:** Characteristics of the study group.

		Study Group	Control Group	*p*
Age [lata] mean ± SD		26.16 ± 5.85	26.09 ± 7.04	0.722 NP
Sex	women [n]	29 (64.44%)	33 (73.33%)	0.495 chi2
	men [n]	16 (35.56%)	12 (26.67%)	

Explanations: p-significance level, Chi2—Chi-square test, NP—non-normality of distribution, non-parametric analysis, Mann–Whitney test, n—number of patients, SD—standard deviation.

**Table 2:** Comparison of individual blood laboratory parameters WBC, OB, CRP.

	Study Group	Control Group	All Patients	*p*
WBC [tys./µL] mean ± SD	6.55 ± 1.45	6.41 ± 1.61	6.48 ± 1.53	0.672 P
OB [mm/h] mean ± SD	6.47 ± 3.4	5.56 ± 3.1	6.01 ± 3.27	0.236 NP
CRP [mg/L] mean ± SD	1.98 ± 1.84	1.88 ± 1.4	1.93 ± 1.63	0.887 NP

Explanations: p—statistical significance, P—normality of distribution, parametric analysis, Student’s *t*-test, NP—non-normality of distribution, non-parametric analysis, Mann–Whitney test, SD—standard deviation.

**Table 3:** Characteristics of the location of lower wisdom teeth and preoperative assessment of the difficulty of its surgical removal according to WHARFE.

		Study Group	Control Group	All Patients	*p*
WHARFE	Mean ± SD	4.82 ± 2.16	5.16 ± 2.03	4.99 ± 2.09	0.299 NP
	median	5	5	5	
	quartiles	3–6	4–6	3–6	
Winter	Distal	7 (15.56%)	6 (13.33%)	13 (14.44%)	0.661 chi2
	Horizontal	10 (22.22%)	8 (17.78%)	18 (20.00%)	
	mesial	20 (44.44%)	18 (40.00%)	38 (42.22%)	
	vertical	8 (17.78%)	13 (28.89%)	21 (23.33%)	

Explanations: p-statistical significance, Chi2—Chi-square test, NP—non-normality of distribution, non-parametric analysis, Mann–Whitney test, SD—standard deviation.

**Table 4:** Pain intensity at six and twelve hours after surgery and postoperative Days 1, 2, 3, 4, 5, 6, and 7 between the study and control groups.

Pain Intensity (0–10)				*p*
		Study Group (*n* = 45)	Control Group (*n* = 45)	
Before surgery	mean ± SD	0 ± 0	0 ± 0	1
	Median (quartiles)	0 (0–0)	0 (0–0)	P
After 6 h	mean ± SD	4 ± 2.08	5.38 ± 2.16	0.003
	Median (quartiles)	4 (2–5)	6 (4–7)	P
After 12 h	mean ± SD	4.31 ± 1.99	5.11 ± 1.87	0.053
	Median (quartiles)	5 (3–6)	5 (4–6)	P
1 day	mean ± SD	3.29 ± 1.87	4.49 ± 1.96	0.006
	Median (quartiles)	4 (2–4)	5 (3–6)	NP
2 days	mean ± SD	2.87 ± 1.6	3.64 ± 1.98	0.077
	Median (quartiles)	3 (2–4)	4 (2–5)	NP
3 days	mean ± SD	2.13 ± 1.59	2.91 ± 1.99	0.039
	Median (quartiles)	2 (1–3)	3 (1–4)	NP
4 days	mean ± SD	1.82 ± 1.56	2.47 ± 1.89	0.096
	Median (quartiles)	2 (1–2)	2 (1–4)	NP
5 days	mean ± SD	1.4 ± 1.45	1.98 ± 1.84	0.169
	Median (quartiles)	1 (0–2)	2 (0–3)	NP
6 days	mean ± SD	1.07 ± 1.29	1.47 ± 1.46	0.178
	Median (quartiles)	1 (0–1)	1 (0–3)	NP
7 days	mean ± SD	0.58 ± 0.75	1.11 ± 1.35	0.123
	Median (quartiles)	0 (0–1)	1 (0–2)	NP

Explanations: p-statistical significance, P—normal distribution in groups, Student’s *t*-test; NP—non-normality of distribution in groups, Mann–Whitney test, n—number of patients, SD—standard deviation.

**Table 5:** Comparison of jaw dilation before surgery and postoperative Days 1, 2, and 7 between the study and control groups.

Trismus		Study Group (*n* = 45)	Control Group (*n* = 45)	*p*
Before surgery	mean ± SD	51.02 ± 6.36	48.08 ± 7.14	0.042
	Median (quartiles)	50.7 (47.06–54.7)	49.26 (43.21–52.05)	P
1 day	mean ± SD	34.23 ± 8.88	27.64 ± 7.44	<0.001
	Median (quartiles)	35 (28–40)	26.52 (22.23–32)	P
2 days	mean ± SD	35.12 ± 9.15	27.68 ± 8.14	<0.001
	Median (quartiles)	34.67 (30–40)	25 (22.04–30.99)	NP
7 days	mean ± SD	43.08 ± 8.35	35.97 ± 8.22	<0.001
	Median (quartiles)	42 (38–47)	36.47 (30–42)	P

Explanations: p—statistical significance, P—normal distribution in groups, Student’s *t*-test; NP-non-normality of distribution in groups, Mann–Whitney test, n—number of patients, SD—standard deviation.

**Table 6:** AB, CD and DE line length measured before surgery and on postoperative Days 1, 2, and 7 between the study group and the control group.

Line AB [cm]		Study Group (*n* = 45)	Control Group (*n* = 45)	*p*
Before surgery	mean ± SD	10.46 ± 0.76	10.62 ± 0.75	0.239
	Median (quartiles)	10.4 (10–11)	10.5 (10–11)	NP
1 day	mean ± SD	10.93 ± 0.81	11.14 ± 1.05	0.298
	Median (quartiles)	11 (10.2–11.5)	11 (10.5–12)	NP
2 days	mean ± SD	10.92 ± 0.79	11.15 ± 0.99	0.23
	Median (quartiles)	11 (10.5–11.5)	11 (10.5–12)	NP
7 days	mean ± SD	10.69 ± 0.83	10.8 ± 0.79	0.517
	Median (quartiles)	10.5 (10–11)	10.8 (10.4–11.5)	P
**Line CD [cm]**		**Study group (*n* = 45)**	**Control group (*n* = 45)**	***p***
Before surgery	mean ± SD	12.03 ± 0.6	12.01 ± 0.73	0.863
	Median (quartiles)	12 (11.5–12.5)	12 (11.5–12.5)	P
1 day	mean ± SD	12.21 ± 0.62	12.26 ± 0.78	0.731
	Median (quartiles)	12.2 (11.8–12.5)	12.2 (12–12.6)	P
2 days	mean ± SD	12.2 ± 0.57	12.28 ± 0.72	0.559
	Median (quartiles)	12.2 (11.8–12.5)	12.2 (11.9–12.8)	P
7 days	mean ± SD	12.07 ± 0.59	12.17 ± 0.71	0.477
	Median (quartiles)	12 (11.5–12.5)	12.2 (11.8–12.5)	NP
**Line DE [cm]**		**Study group (*n* = 45)**	**Control group (*n* = 45)**	***p***
Before surgery	mean ± SD	11.69 ± 0.72	11.72 ± 0.89	0.846
1 day	Median (quartiles)	11.5 (11.3–12)	11.5 (11–12.4)	P
	mean ± SD	12.03 ± 0.67	12.1 ± 0.78	0.666
2 days	Median (quartiles)	12 (11.5–12.5)	12 (11.5–12.5)	P
	mean ± SD	12.1 ± 0.73	12.22 ± 0.79	0.438
7 days	Median (quartiles)	12 (11.5–12.5)	12.2 (11.6–13)	P
	mean ± SD	11.8 ± 0.69	11.92 ± 0.87	0.494
	Median (quartiles)	11.8 (11.4–12)	12 (11.5–12.5)	P

Explanations: p—statistical significance, P—normal distribution among groups, Student’s *t*-test; NP—non-normality of distribution among groups, Mann–Whitney test, n—number of patients, SD—standard deviation.

**Table 7:** Comparison of temperature values measured before surgery and on the 1st, 2nd, and 7th post-surgery days between the study and control groups.

Body Temperature [°C]		Study Group (*n* = 45)	Control Group (*n* = 45)	*p*
Before surgery	mean ± SD	36.57 ± 0.28	36.66 ± 0.25	0.196
	Median (quartiles)	36.6 (36.4–36.8)	36.6 (36.5–36.8)	NP
1 day	mean ± SD	36.6 ± 0.45	36.76 ± 0.47	0.103
	Median (quartiles)	36.6 (36.2–36.9)	36.6 (36.6–37)	NP
2 days	mean ± SD	36.52 ± 0.39	36.74 ± 0.47	0.022
	Median (quartiles)	36.5 (36.2–36.8)	36.7 (36.4–37)	NP
7 days	mean ± SD	36.58 ± 0.4	36.7 ± 0.41	0.14
	Median (quartiles)	36.5 (36.3–36.8)	36.6 (36.5–36.9)	NP
**Surgery site temperature [°C]**		**Study group (*n* = 45)**	**Control group (*n* = 45)**	***p***
Before surgery	mean ± SD	38.08 ± 0.7	38.27 ± 0.53	0.17
	Median (quartiles)	38.3 (37.7–38.5)	38.4 (38.1–38.7)	NP
1 day	mean ± SD	38.52 ± 0.54	38.56 ± 0.47	0.665
	Median (quartiles)	38.5 (38.3–38.9)	38.4 (38.3–38.9)	P
2 days	mean ± SD	38.54 ± 0.45	38.38 ± 0.38	0.086
	Median (quartiles)	38.5 (38.2–38.8)	38.4 (38.2–38.6)	P
7 days	mean ± SD	38.38 ± 0.43	38.28 ± 0.48	0.313
	Median (quartiles)	38.4 (38.1–38.7)	38.3 (38.1–38.6)	P

Explanations: p—statistical significance, P—normal distribution in groups, Student’s *t*-test, NP—non-normality of distribution in groups, Mann–Whitney test, n—number of patients, SD—standard deviation.

## Data Availability

Data available on request.
